# Strengthening the scale‐up and uptake of effective interventions for sex workers for population impact in Zimbabwe

**DOI:** 10.1002/jia2.25320

**Published:** 2019-07-22

**Authors:** Frances M Cowan, Sungai T Chabata, Sithembile Musemburi, Elizabeth Fearon, Calum Davey, Tendayi Ndori‐Mharadze, Loveleen Bansi‐Matharu, Valentina Cambiano, Richard Steen, Joanna Busza, Raymond Yekeye, Owen Mugurungi, James R Hargreaves, Andrew N Phillips

**Affiliations:** ^1^ Department of International Public Health Liverpool School of Medicine Liverpool United Kingdom; ^2^ Centre for Sexual Health and HIV AIDS Research (CSHHAR) Zimbabwe Harare Zimbabwe; ^3^ Department of Population Health London School of Hygiene and Tropical Medicine London United Kingdom; ^4^ Institute for Global Health University College London London United Kingdom; ^5^ Department of Public Health Erasmus University Rotterdam The Netherlands; ^6^ National AIDS Council Harare Zimbabwe; ^7^ AIDS and TB Directorate Ministry of Health and Child Care Harare Zimbabwe

**Keywords:** sex workers, HIV infection, Zimbabwe, Africa, HIV prevention, HIV testing

## Abstract

**Introduction:**

UNAIDS’ goal of ending AIDS by 2030 is unreachable without better targeting of testing, prevention and care. Female sex workers (FSW) in Zimbabwe are at high risk of HIV acquisition and transmission. Here, we report on collated programme and research data from Zimbabwe's national sex work programme. We also assess the potential for wider population impact of FSW programmes by modelling the impact on HIV incidence of eliminating transmission through FSW (i.e. calculate the population attributable fraction of incidence attributable to sex work).

**Methods:**

Descriptive analyses of individual‐level programme data collected from FSW between 2009 and June 2018 are triangulated with data collected through 37 respondent driven sampling surveys from 19 sites in Zimbabwe 2011 to 2017. We describe programme coverage, uptake, retention and patterns of sex work behaviour and gaps in service provision. An individual‐level stochastic simulation model is used to reconstruct the epidemic and then the incidence compared with the counter‐factual trend in incidence from 2010 had transmission through sex work been eliminated from that date.

**Results:**

Sisters has reached >67,000 FSW since 2009, increasing attendance as number of sites, programme staff and peer educators were increased. Over 57% of all FSW estimated to be working in Zimbabwe in 2017 (n = 40,000) attended the programme at least once. The proportion of young FSW reached has increased with introduction of the “Young Sisters programme.” There are no clear differences in pattern of sex work across settings. Almost all women report condom use with clients at last sex (95%); however, consistent condom use with clients in the last month varies from 52% to 95% by site. Knowledge of HIV‐positive status has increased from 48 to 78% between 2011 and 2016, as has prevalence of ART use among diagnosed women (29 to 67%). Although subject to uncertainty, modelling suggests that 70% (90% range: 32%, 93%) of all new infections in Zimbabwe from 2010 are directly or indirectly attributable to transmission via sex work.

**Conclusions:**

It is feasible to increase coverage and impact of sex work programming through community‐led scale‐up of evidence‐based interventions. Eliminating transmission through commercial sex would likely have a substantial impact on new infections occurring more widely across Zimbabwe.

## 1 Introduction

The UNAIDS goal of eliminating HIV by 2030 is unreachable without targeting testing, prevention and care 1, 2 more effectively. Despite 21 of 37 million people living with HIV (PLHIV) accessing antiretroviral therapy (ART) globally, 1.8 million new infections occurred in 2017, 980,000 in sub‐Saharan Africa. The region's demographic transition, wherein 40% of the region's population is aged <15 years means that without substantial reductions in HIV incidence, the absolute number of new HIV infections in southern Africa will double in the next 20 years 3, 4.

These figures hide disparity in risk of HIV acquisition by age, sex, geography and behaviour 1. For example, odds of HIV infection among female sex workers (FSW) across sub‐Saharan Africa (SSA) is 11 times higher than among adult women generally 5. Suboptimal care‐seeking among FSW is associated with poor health outcomes 6, contributing a substantial proportion of new infections in the broader population 7, 8. Modelling studies from around Africa suggest that previous assessments of transmission, which did not fully take into account indirect transmissions, greatly underestimated the likely contribution of sex work even in the generalized epidemics of SSA 7, 8. Furthermore, as treatment has become more widely available, thus reducing transmissions within the general population, the relative importance of transmission from commercial sex to overall numbers of new infections may well have increased. Sex work interventions are highly cost effective 9 and calls to take programmes to scale have intensified in recent years 10, 11.

Optimizing FSW programming requires maximizing FSW ownership and uptake of and engagement in prevention and treatment cascades 1, 12, 13. Programmes need to build community empowerment to ensure that effective interventions (HIV testing, treatment for sexually transmitted infections, treatment for HIV‐infected FSW, consistent condom use and pre‐exposure prophylaxis (PrEP)) are taken to scale with sufficient intensity, and that they include the most vulnerable and difficult to reach, that is, young women 14; recent entrants into sex work; and those with highest client load, inconsistent condom use and problematic drug or alcohol use 15.

In this paper, we collate programme and research evidence from Zimbabwe's national sex work programme “Sisters with a Voice” to (1) present the current impact that engagement in the Sisters programme has on HIV incidence, prevalence and control in FSW (based on programme and respondent driven sampling (RDS) survey data), (2) to describe the patterns and characteristics of sex work among FSW in Zimbabwe (based on RDS survey data), and (3) to assess the potential for wider population impact of sex worker programmes by modelling the impact on HIV incidence of eliminating transmission through FSW (i.e. calculate the population attributable fraction of incidence attributable to sex work).

## 2 Methods

### 2.1 Sisters with a Voice programme

#### 2.1.1 Overview of the programme

**Figure 1:**
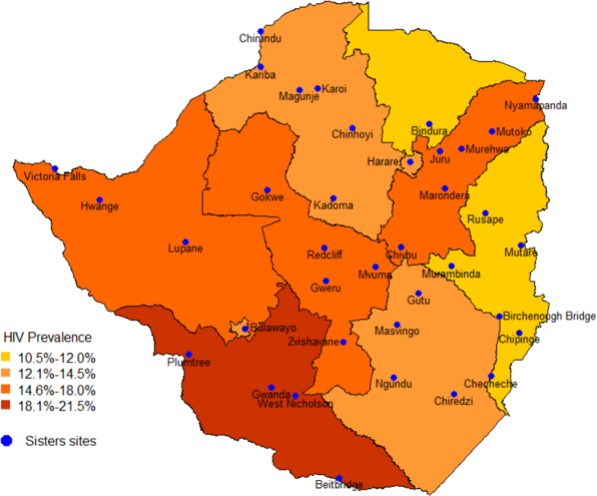
Map of general population HIV prevalence by province with location of Sisters sites superimposed.

Zimbabwe's national sex work programme, Sisters with a Voice, was established on behalf of Ministry of Health and Child Care and National AIDS Council in 2009. The programme expanded from five initial sites to 13 (2010) and then 36 sites (2013 to 2017), although five small sites recently closed due to funding constraints. Sites are located at major urban, town and highway hubs for sex work transmission (see Figure 1).

#### 2.1.2 Data collection and analysis

Since the start of the programme, comprehensive data have been collected from all women seeking clinical services and more recently (2016), from women engaged with community‐based peer education.

At the first visit to the Sisters programme, women are assigned an alphanumeric unique identifier. Each time a woman visits the programme she is asked if she has attended previously and if so, her records are retrieved. The ID links consultations within and across sites, except where women provide false information. Prior to 2013, data were collected manually by nursing staff and single‐entered into a Microsoft Access database. At all visits, information was collected on whether FSW had ever tested for HIV and the date and result of the most recent test, some self‐reported and some conducted and recorded by the programme. Among women identified as living with HIV, data were collected at each visit on whether and when antiretroviral drugs (ARV) had been initiated and if these were currently being taken (although the Sisters programme does not itself provide ART). Since 2013 the same data have been collected using real time electronic data capture synced daily to secure cloud storage.

We estimated the proportion of FSW “in the programme” who for each month since 2009 fell into one of five statuses: (1) Never tested for HIV; (2) HIV negative and not tested in the last six months; (3) HIV negative and tested in the last six months; (4) HIV positive and not on ART; (5) HIV positive and on ART. FSW were considered to be “in the programme” at a given time point if they had (1) had a visit within the last six months or (2) had a visit more than six months prior and in addition have another visit recorded at a future timepoint, beyond 31 December 2017. We used both self‐reported HIV testing data and results and HIV tests conducted by the programme.

#### 2.1.3 Research data

Research has been integral to the development, expansion and evaluation of the programme, with respondent driven sampling surveys conducted through three studies conducted at 19 sites: in 2011 and 2015 (repeat surveys conducted in three sites), 2013 and 2016 (repeat surveys conducted in 14 sites including one site surveyed in 2011/15 surveys) and 2017 (three additional survey sites including Harare and Bulawayo), showing a mean HIV prevalence of 58% (range 44% to 82%) 16, 17, 18, 19. HIV incidence has been estimated from analysis of programme data between 2009 to 2013 at 10% per annum and 7% in 18 to 24 year olds 20, 21.

#### 2.1.4 Synthesis of research data

Data from the three RDS survey studies conducted in 19 sites 19, 22, 23 (17 on highways or in towns around mines, army camps plus Harare and Bulawayo) provide a picture of sex work in Zimbabwe. In all surveys, eligible FSW had exchanged sex for money in the past 30 days, were aged ≥18, and had been working in the interview site for at least six months (30 days for Harare, Bulawayo and one rural site). In each site, we conducted mapping, followed by purposive selection of “seeds” representing a mix of ages, sex work types and geographic locations. We interviewed seeds and gave them two coupons to distribute to peers. Women receiving a coupon could attend an interview and were subsequently given two coupons for their peers. Five to seven iterations of this process (“waves”) were performed, excluding the seeds. Participants were given US$5 compensation for their time, and US$2 for each participant recruited. Checks were included to ensure coupons were genuine and minimize repeat participation. Interviewer‐administered questionnaire data were collected on tablet computers and included demographics, sex work, sexual behaviour, HIV prevention and care uptake and on personal network size for RDS adjustment. A finger‐prick blood‐sample was collected from each FSW for HIV antibody testing, and for two studies (17 sites), measurement of HIV viral load.

Relevant data from the surveys were merged and patterns of sex work and related behaviours and treatment cascade indicators analysed descriptively across surveys. We explored differences by type of Sisters site (rural, highway, town, city, Bulawayo and Harare). Although women were recruited anonymously, all provided information combining initials and date of birth to create a “check‐identifier” to minimize risk of duplicate enrolment. Within the combined dataset we identified seven participants who had similar “check‐identifiers” but their questionnaire responses suggested they were different FSW. One FSW likely took part in surveys at two different sites. As the check‐identifier was not “fail safe” data were retained from her participation in both surveys. We looked at changes in HIV cascade indicators among 2011, 2013 and 2015/6/7.

Our approach to RDS analysis has been described in detail elsewhere 19, 22. We followed guidance to assess evidence of bias in our operationalization of RDS 24. We used the RDS‐II method for analysis: dropping seed responses and weighting each woman in each site by the inverse of her network size, that is, the number of other women she could have recruited 25. Data were pooled across surveys and weighted for size of site (not study) and weighted using site‐normalized inverse degree weights. All analyses were at the cluster (site) level.

### 2.2 Modelling

#### 2.2.1 Overview of the existing HIV synthesis model

The HIV Synthesis model has been used to assess (potential) effectiveness and/or cost effectiveness of a range of interventions in SSA, including HIV self‐testing 26, a vaccine 27, a cure 28, ART monitoring 29, 30 and drug resistance testing 31. It is an individual‐based model of HIV transmission, progression and the effect of ART in adults. Each time the model is run it simulates values of variables for the number of short term condomless sex partners, presence of a long term condomless sex partner, HIV testing, HIV acquisition and additionally, in PLHIV, viral load, CD4 count, use of specific ART drugs, adherence to ART, resistance to specific drugs and risk of HIV‐related death, each updated in three‐month time steps from 1989. It is informed by extensive cohort and survey data from within and outside Zimbabwe. A woman is designated a condomless sex FSW if she has at least three condomless sex partners in a three‐month period in the past year. We further consider that a proportion of women are FSW but that they always use condoms (and thus have no possibility of acquisition or transmission of HIV) 32.

#### 2.2.2 Calibration of the model

For this paper, the model was calibrated to data from Zimbabwe. Data items to which the model is calibrated are HIV prevalence, HIV incidence, number of HIV tests performed per year, PLHIV with known status, number receiving ART, number on second line, proportion with viral suppression, proportion with resistance, number of pregnant women and number of FSW 32. The data from RDS surveys are used to inform the calibration in respect of the number of FSW as well as prevalence among FSW and we have further compared the model outputs with observed sex worker programme data on the cascade of HIV care 32.

### 2.3 Ethical issues

All research studies had ethical approval from the Medical Research Council of Zimbabwe (MRCZ) and collaborating universities (University College London, London School of Hygiene and Tropical Medicine and Liverpool School of Tropical Medicine). All participants provided written informed consent obtained according to principles of good clinical practice. Approval of secondary data analysis of programme data has been provided by MRCZ.

## 3 Results

### 3.1 Programme attendance

**Figure 2:**
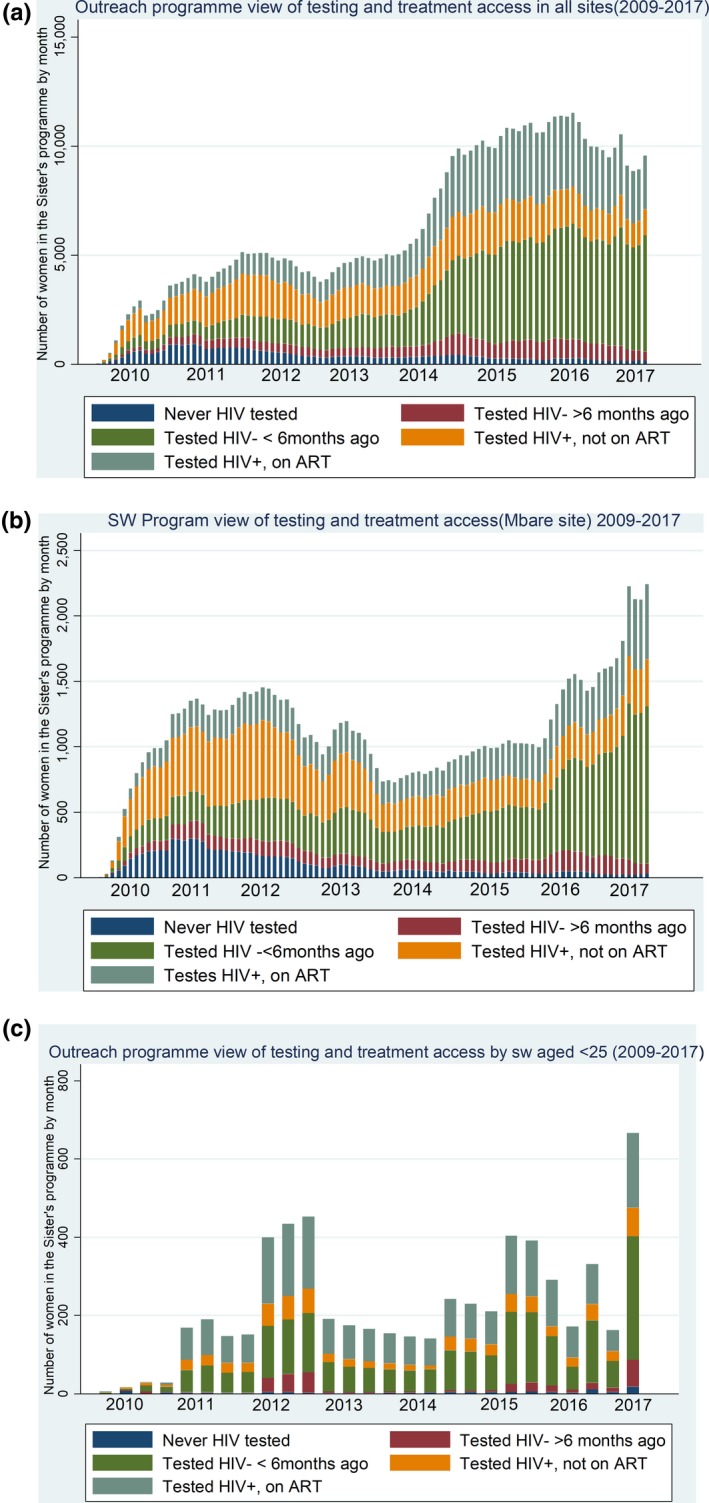
Outreach programme view of testing and treatment access in (a) all sites 2009 to 2017 (b) Mbare, Harare 2009 to 2017. (c) all sites by FSW aged <25 years 2009 to 2017.

By September 2018 over 67,000 women had been seen at least once, for 194,000 clinic visits. As service provision is scaled up, the number of women reached (and reached repeatedly) increases (see Table 1, Figure 2a). The proportion of young women has increased since the development of the “Young Sisters” programme in 2014 supported by teen peer educators 33 and scaled up across the programme from 2016. In 2017, the last year for which full statistics are available, over 24,000 women were reached with clinical services (57% of all FSW estimated in Zimbabwe). Self‐reported condom use has increased over time. In 2017, 65% of attendees reported condom use at last sex and 52% reported consistent condom use with all clients in the past month. Of note, services intensified in the major cities during 2017, peer educator numbers increased, including those aged <25 from 13 to over 70, allowing more intensive outreach within the cities. This had an impact on number of FSW reached and the proportion of young women reached (see Figure 2b for FSW of all ages in Harare and 2c for FSW <25 for sites overall).

**Table 1:** Sisters programme implementation by year showing number of clinics operating, intensity of clinical service provision, numbers and age of peer educators

Year of program operation	# Static sites^a^	# Out‐reach sites^b^	# Peer educators	# Sites young sisters program	# Peer educators aged <25	# FSW seen	% FSW < 25	% FSW < 20	# FSW seen first time	% FSW first time <25	% First time FSW < 20	# Reached by community mobilization	% FSW aware of HIV status^c^	% Consistent condom use last month	% Condom use at last sex with client	Comment on implementation
2009	1	4^d^	41	0	NA	322	25.8	6.5	322	25.8	6.5	NA	23.9	38.4	NA	
2010	3	13^d^	131	0	NA	3394	22.3	5.8	3196	22.2	5.9	NA	44.6	35.5	NA	
2011	3	13^d^	131	0	NA	5083	21.0	5.8	3642	22.2	6.4	NA	58.9	35.4	40.6	
2012	3	13^d^	131	0	NA	4021	22.3	7.4	2442	25.1	8.9	NA	67.1	44.7	69.8	Funding interrupted for four months
2013	6	27^d^	100	0	NA	6151	23.7	7.3	4255	25.0	7.8	NA	69.6	35.1	57.7	Election year slowed programme implementation
2014	6	30^e^	170	3	NA	13,045	23.7	7.8	9895	25.9	8.8	4560	72.8	28.0	69.8	
2015	6	30^e^	170	3	NA	16,688	23.8	7.5	10,539	27.8	9.5	17,186	75.9	24.2	74.3	
2016	6	30^e^	184	9	54	18,539	25.5	7.8	10,657	32.9	11.5	21,885	75.3	51.4	70.1	Funding to 18 outreach sites withdrawn for four months
2017	6	30^e^	280	24	90	24,563	30.0	11.7	15,638	38.5	16.7	13,348	69.3	51.9	64.6	Supplemental funding for activities in five main cities
2018	10	20^e^	191	30	65	10,731	28.2	9.5	5066	38.4	15.0	2485	73.7	59.4	64.8	January to June only. Delayed implementation – donor funding transition

^a^static sites sited in government clinics, open all day Monday to ‐Friday; ^b^outreach sites in small towns, mines highways, sited in government clinics; ^c^aware of HIV positive status or tested HIV negative within six months when attended clinic; ^d^open one day each fortnight. Peer educators on site in between; ^e^open one day per week, peer educators on site in between.

In Figures 2a,b,c, the pattern of engagement with Sisters services is shown over time. While attendance has increased overall (Figure 2a), funding gaps led to temporary reductions in service provision. There was a six‐month funding gap in the second and third quarters of 2012. 2013 was an election year, resulting in some temporary clinic closures. In 2014, funding increased, allowing 30 Sisters outreach sites to be opened weekly instead of bimonthly, but at the end of 2016 and again in 2017 18 outreach sites were closed due to funding restrictions.

In Harare (Figure 2b) the Sisters clinic was moved out of the City of Harare (Mbare) clinic and into the Central Business District between 2013 and 2016 due to space concerns, reducing acceptability of the clinic to FSW. Attendance returned to previous levels when the clinic relocated back to Mbare. When services in Harare were intensified in 2016/7 there was greatly increased attendance. Recruiting younger peer educators and specifically targeting younger FSW has resulted in an increase in the number and proportion of younger women seen.

The increased engagement of FSW attending the Sisters programme with testing and treatment is striking (Figures 2a,b,c). In 2009, the majority of those diagnosed HIV positive were not on treatment (eligibility for ART was determined by CD4 count), and around one‐third had never tested for HIV. By 2017, almost all had tested previously, among those who previously tested negative the majority had tested within the last six months, and among those testing positive an increasing proportion were on ART across all sites.

### 3.2 Pattern of Sex Work

A total of 6096 women were recruited into 3 studies at 19 sites between 2011 and 2017. Table 2 shows their characteristics and patterns of work by location.

**Table 2:** Characteristics of female sex workers and their pattern of work by type of geographic location

	Rural (3 sites) (N = 609) n (%)	Highway (3 sites) (N = 615) n (%)	Towns (9 sites) (N = 1953) n (%)	Cities (2 sites) (N = 614) n (%)	Bulawayo (N = 808) n (%)	Harare (N = 1497) n (%)
Age (years)						
Mean (SD)	33 (9.4)	32 (8.6)	32 (8.5)	34 (9.7)	32 (9.3)	31 (8.0)
Median (IQR)	32 (11)	31 (11)	31 (11)	33 (14)	31 (13)	31 (11)
Education						
None	28 (3.7)	18 (3.9)	64 (2.4)	19 (2.6)	7 (0.7)	28 (1.8)
Primary	188 (40.3)	150 (26.3)	544 (27.6)	169 (22.8)	186 (24.2)	410 (28.9)
Secondary	387 (55.8)	444 (69.5)	1339 (70.0)	424 (74.3)	607 (73.8)	1056 (69.1)
Tertiary	6 (0.2)	2 (0.3)	6 (0.1)	2 (0.3)	8 (1.3)	2 (0.2)
Marital status						
Divorced/separated	408 (78.9)	434 (71.6)	1226 (63.0)	370 (58.6)	394 (48.1)	1026 (70.3)
Widowed	136 (17.6)	99 (15.6)	358 (12.0)	130 (19.2)	108 (13.1)	217 (13.8)
Never been married	63 (3.4)	78 (12.4)	335 (23.7)	109 (21.7)	285 (36.7)	240 (14.8)
Married/cohabiting	2 (0.1)	4 (0.4)	33 (1.3)	5 (0.5)	21 (2.1)	14 (1.1)
Number of children <18 years						
0	93 (17.2)	100 (15.5)	306 (17.2)	72 (11.6)	158 (23.4)	196 (12.9)
1 to 2	313 (46.1)	283 (47.0)	1065 (58.1)	348 (61.7)	367 (43.2)	765 (53.9)
≥3	203 (36.7)	232 (37.5)	582 (24.7)	194 (26.7)	283 (33.4)	536 (33.2)
Women aged <25 years with dependent children						
No	25 (23.6)	36 (30.3)	92 (23.3)	22 (15.0)	79 (42.4)	72 (20.1)
Yes	80 (76.4)	79 (69.7)	270 (76.7)	91 (85.0)	140 (57.6)	140 (79.9)
Age at first selling sex (years)						
Mean (SD)	26 (7.7)	26 (39.8)	26 (23.1)	25 (7.2)	24 (7.2)	25 (6.6)
Median (IQR)	25 (10)	24 (8)	24 (9)	24 (9)	23 (10)	24 (9)
Duration in sex work (years)						
Mean (SD)	8 (6.8)	8 (6.6)	7 (6.4)	9 (7.7)	7 (7.3)	7 (5.7)
Median (IQR)	5 (7)	5 (8)	5 (7)	6 (10)	5 (8)	5 (6)
Venue for client recruitment						
Bars/nightclubs/other venue	342 (80.9)	293 (56.9)	1365 (81.5)	411 (71.6)	675 (84.8)	939 (63.7)
By telephone	24 (1.6)	37 (6.1)	120 (13.8)	50 (12.1)	22 (2.1)	12 (0.8)
In the market place/street	126 (16.0)	143 (26.7)	252 (4.4)	71 (16.0)	89 (11.2)	501 (32.8)
Other	98 (1.5)	58 (10.3)	139 (0.3)	15 (0.3)	21 (1.9)	39 (2.7)
Number of clients in the last week						
Mean (SD)	7 (8.2)	8 (8.2)	8 (8.7)	12 (13.5)	8 (8.3)	14 (15.4)
Median (IQR)	5 (7)	5 (7)	5 (7)	7 (11)	5 (7)	10 (15)
Currently have a steady partner						
No	221 (45.1)	206 (31.9)	722 (23.9)	286 (49.7)	262 (33.3)	793 (52.8)
Yes	388 (54.9)	409 (68.1)	1231 (76.1)	328 (50.3)	546 (66.7)	704 (47.2)
Price per client – short time						
<$5	548 (93.5)	471 (76.6)	1218 (24.3)	359 (55.3)	596 (74.7)	1389 (93.8)
>$5 to $10	56 (6.2)	127 (21.1)	616 (68.8)	169 (36.5)	196 (24.0)	91 (5.2)
>$10 to $20	2 (0.2)	11 (2.1)	47 (6.2)	28 (7.0)	11 (1.3)	15 (1.0)
>$20	1 (0.1)	1 (0.2)	8 (0.7)	2 (1.2)	0 (0.0)	0 (0.0)
Proportion of income generated through sex work						
<25%	46 (9.6)	31 (4.2)	133 (2.4)	39 (2.4)	86 (6.6)	238 (14.9)
25% to 50%	103 (6.7)	108 (16.2)	255 (11.2)	75 (13.0)	80 (7.9)	210 (14.1)
>50% to 99%	195 (13.6)	233 (39.6)	528 (27.8)	177 (32.4)	173 (21.7)	258 (17.9)
100%	265 (70.1)	243 (40.0)	1031 (58.6)	469 (52.2)	469 (63.8)	791 (53.1)
Relationship with other female sex workers						
Good	425 (57.6)	429 (72.2)	1294 (67.7)	454 (72.5)	559 (66.5)	559 (58.2)
Neither good nor bad	147 (36.2)	160 (24.3)	489 (20.9)	121 (19.6)	208 (28.8)	208 (33.8)
Bad or no relationship	36 (6.2)	26 (3.4)	168 (11.4)	39 (7.9)	41 (4.7)	41 (8.0)
Frequency of alcohol use in past 12 months						
Never	216 (28.1)	256 (42.6)	692 (33.8)	194 (29.8)	172 (21.4)	406 (26.5)
Once a month or less	71 (15.2)	66 (9.2)	191 (9.2)	84 (19.5)	70 (8.9)	165 (12.2)
2 to 4 times per month	54 (6.3)	61 (9.5)	312 (20.7)	129 (22.3)	87 (12.1)	185 (13.5)
2 to 3 times per week	124 (25.9)	121 (21.0)	334 (15.4)	82 (12.4)	261 (33.8)	399 (26.6)
4 or more times per week	144 (24.5)	111 (17.7)	421 (20.9)	125 (16.0)	125 (23.8)	342 (21.2)
Experience of physical violence from steady partner						
No	322 (46.9)	314 (49.5)	1055 (64.2)	327 (52.4)	422 (58.4)	708 (47.1)
Yes	287 (53.1)	301 (50.5)	898 (35.8)	287 (47.6)	386 (41.6)	789 (52.9)
Experience of physical violence from client						
No	464 (72.0)	433 (72.2)	1432 (82.5)	450 (76.1)	566 (74.9)	952 (66.2)
Yes	145 (28.0)	182 (27.8)	520 (17.5)	164 (23.9)	242 (25.1)	545 (33.8)
Ever been raped						
No	570 (72.0)	550 (72.2)	1805 (82.5)	581 (76.1)	690 (74.9)	1225 (66.2)
Yes	39 (28.0)	65 (27.8)	148 (17.5)	31 (23.9)	118 (25.1)	272 (27.8)
Experience of violence from police in the past 12 months						
No	568 (97.1)	547 (91.7)	1671 (93.0)	550 (92.3)	729 (90.3)	1297 (87.4)
Yes	40 (2.9)	67 (8.3)	265 (7.0)	63 (7.7)	78 (9.7)	196 (12.6)
Condom use at last sex with client						
No	26 (5.2)	20 (4.1)	57 (2.9)	28 (5.7)	31 (4.9)	57 (3.5)
Yes	583 (94.8)	595 (95.9)	1885 (97.1)	584 (94.3)	776 (95.1)	1439 (96.5)
Condom‐less sex with client in the past month						
No	400 (93.4)	333 (52.4)	1117 (70.9)	384 (72.6)	670 (83.0)	1255 (81.5)
Yes	173 (6.6)	266 (47.6)	684 (29.1)	220 (27.4)	138 (17.0)	242 (18.5)

Between 20% and 28% of FSW were aged <25 and the majority had some education; those from rural areas were less educated. Most had dependent children and the majority in all sites other than Bulawayo were divorced or separated. In terms of behaviour, 11% to 19% started selling sex below age 18 (defined as child abuse by UN agencies), 7% to 19% had worked as FSW for less than a year, most meet clients in bars or on the street, and 14% to 36% had over 10 clients in the previous week. Women typically received under US$5 per sex act although those in towns (but not cities) more often reported receiving US$5‐10. There are some differences in characteristics/behaviours among FSW working in different settings but no clear patterns.

**Figure 3:**
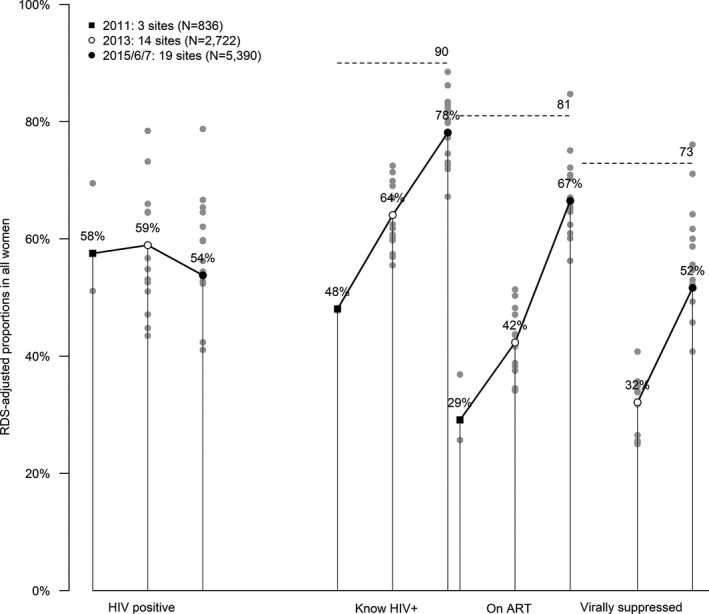
Engagement of female sex workers in the HIV care cascade 2011, 2013, 2015/6/17. The treatment cascades for the three time point arms are illustrated, with the left of each bar showing results for 2011, the middle for 2013 and the right 2016. The cascades are shown for all women included in the relevant surveys, with the 90:90:90 targets indicated with horizontal dotted lines. All values were adjusted with RDS‐II. The dots represent the value of cascade indicator for each site surveyed in that year.

Data on service use across the 17 sites that have data for all cascade steps are presented in Figure 3. Engagement with care has increased over time. In 2015/6/7 FSW LHIV, 78% were aware of their diagnosis, 85% of those reported being on ART and 82% of those reporting ART were virologically suppressed. Among FSW LHIV overall 66% had a viral load <1000 copies/mL. Among HIV negative FSW, 76% reported having tested within the preceding six months.

### 3.3 Using modelling to predict potential for averting new infections through targeting FSW

**Figure 4:**
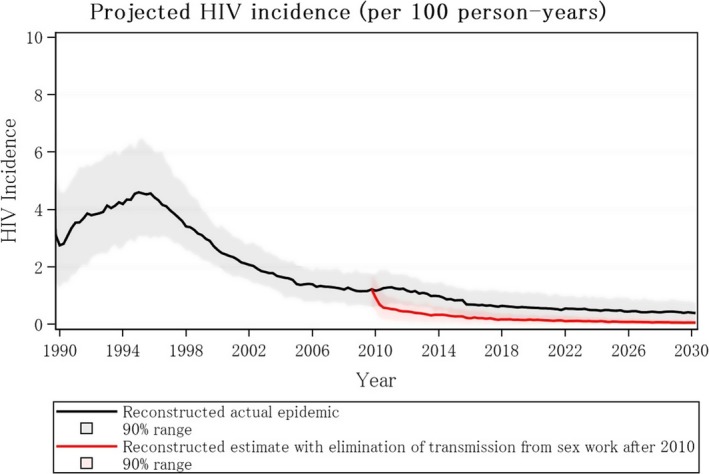
Projected HIV incidence among general population in Zimbabwe with (red line) and without (black line) elimination of transmission in either direction between sex workers and clients from 2010.

Figure 4 presents historical data and projections of HIV incidence with and without condomless transactional sex after 2010. This was done by first reconstructing the epidemic as it has played out and then re‐running the re‐construction but eliminating condomless sex work, based on the assumption that condomless sex work corresponds to a woman having more than three different condomless partners in a three‐month period. It thus involves assuming that all women with multiple partners at this level or beyond are FSW 34. Figure 4 indicates the potential course of the HIV epidemic had transmission via sex work ceased from 2010. The comparator (black line) conveys the actual epidemic as it has played out and takes into account the increased prevention, diagnosis and successful use of ART in this period. Overall there is an estimated 70% (90% uncertainty bound 32% to 93%) of infections attributable to sex work. In this analysis model the maximum number of FSW in 2017 was 203,000 (median 116,000 FSW, median 47,000 condomless sex FSW). If we restrict to runs for which the number of FSW is below 110,000 (median number of FSW 90,000, condomless sex FSW 39,000), an estimated 72% (90% uncertainty bound 32% to 89%) of infections were attributable to sex work.

Moreover, compared with a scenario in which the epidemic and programme continues as currently, with the same rate of HIV testing, ART initiation and retention, current rate of circumcision to 2030, there would be 85% less new infections in 2030 had transmission through sex work been eliminated from 2010.

## 4 Discussion

We describe trends over time in key characteristics of FSW engagement in services in the context of a national FSW programme supported by peer‐led mobilization and empowerment and general scale up of ART. These suggest the potential feasibility of virtually eliminating HIV transmissions through commercial sex in Zimbabwe. We show that this would likely have substantial impact on the Zimbabwe epidemic more generally. Increasing coverage and intensity of service delivery by strengthening community involvement and empowerment alongside increasing numbers of programme staff led to concomitant increases in coverage, status awareness and uptake and optimal use of ART. In our recently published SAPPH‐IRe trial of a targeted combination prevention intervention between 2013 and 2016 22 there were substantial gains in engagement in both arms; more intensive service provision in the intervention arm was associated with greater community mobilization of women (twice as many new HIV infections were diagnosed), although there was no significant difference by arm in the proportion of all FSW with VL <1000 copies/mL. The proportion of HIV positive FSW with VL <1000 copies/mL rose from 50% in 2013 to 68% to 73% in 2016, suggesting that increasing FSW programme coverage and intensity in the context of a well‐functioning national ART programme can have a substantial impact on the proportion of FSW with infectious HIV. Of note, the rate of virological suppression at endline in the SAPPH‐IRe trial compares favourably with data from ZIMPHIA which found that 65% of all HIV positive women were virologically suppressed 35.

Community empowerment of FSW is a critical component of effective programming and has been UNAIDS best practice for well over a decade 36. Optimizing programme impact requires that empowered FSW know about and are motivated to use services, that there are acceptable, accessible comprehensive services in place and FSW accessing these services are supported to fully engage with them 12. We can consider the extent of programme activity in the context of an estimate of the size of the FSW population in Zimbabwe. In a separate study, we have combined size estimation data from 20 sites and extrapolated from programme data in a further 16**,** to estimate that there are 40,000 (plausibility bounds 28,000 to 59,000) women working as sex workers in Zimbabwe (1.23% (plausibility bounds 0.86% to 1.79%) of adult female population (Fearon *et al*., unpublished data) with around 20,000 (50%) in Harare and Bulawayo. In 2017, over 24,000 FSW engaged with the national programme, 60% of the estimated sex work population; 8677 were in Harare and Bulawayo, which is 43% of the estimated populations in those cities.

Although attendees’ mean number of clinical contacts with the programme has increased to three, it falls short of quarterly visits recommended by WHO 37. If the “optimal ratio” of peer educator to FSW is 1:50 to provide sufficient community support for service engagement 38, for example, then 280 peer educators rather than the current 70 are required in Harare. Of note, a government, donor and implementor panel convened in 2017 to inform national size estimation estimated that the location of Sisters programme sites provides the potential for access to between 75% and 85% of all FSW nationally (Fearon *et al*., unpublished data), although since then six sites have closed due to funding constraints. Thus, extending the capacity of existing sites plus establishing new sites at hotspots not currently covered is needed. In addition, training of health care providers within government services to ensure that they are FSW friendly is critical. As condomless sex is commonly reported (and more commonly with regular partners and among programme attendees, perhaps reflecting less social desirability bias in this setting) 39 scaling and supporting optimal use of PrEP is increasingly urgent; proportionately few HIV negative FSW (<15% by July 2018) are on PrEP (personal communication Emily Gwavava). Extending services to ensure that regular partners of FSW can access HIV and STI testing and treatment as well as condoms is critical.

Intensifying community engagement and empowerment, using microplanning, and initiating self‐help groups to build trust, social cohesion and community ownership (with the aim that self help groups transition over time to become FSW‐led community‐based organizations) could further increase effectiveness of prevention and care programmes 12, 40, 41. Peer‐led microplanning, which provides regular, risk‐differentiated, individual peer support has potential to improve service uptake, including regular repeat HIV testing, and adherence to PrEP and ART. Despite considerable gains, further scale‐up and intensification of targeted programming in partnership with FSW is needed to maximize impact and likely cost effectiveness.

This study has many strengths. It triangulates individual‐level programme data from over 67,000 FSW collected over nine years with representative survey data from across Zimbabwe between 2011 and 2017 plus other research data. This allows us to convincingly show that with increasing coverage and intensity of service provision supported by increasing community ownership, HIV status awareness and engagement with the treatment cascade has improved. In addition, these analyses have allowed us to identify gaps in service provision, uptake and use that can be targeted. These data have been used to inform the HIV Synthesis model for Zimbabwe, so we can assess in broad terms the potential impact of eliminating direct and indirect acquisition and transmission of HIV resulting from commercial sex transactions in Zimbabwe and the region more broadly. We plan to further develop the model to inform programme targeting and regional policy.

Study limitations include the self‐reporting of testing and ART initiation in programme and survey data, and some 10% of programme variables are missing. Our use of respondent‐driven sampling to recruit FSW might have been subject to bias. Refusal rates are difficult to document with this design. Our analyses suggested little evidence of bias, but were not definitive. We have not thus far been able to identify longitudinal data on frequency of transitions into and out of sex work, and little is known about the life course of sex work and how this varies according to sex work typologies that can be reflected in the model. We are not clear on what proportion of all women with three or more condomless sex partners in a three‐month period identify as FSW, who the others are, and how to reach them. For this reason, our estimate of 70% of new infections after 2010 attributable to sex work is associated with considerable uncertainty, even beyond that captured by the uncertainty bounds we present which represent variation over model runs with different input parameter values. Nevertheless, there is little uncertainty over the fact that this attributable fraction is substantial. Additionally, our model does not explore differentiated targeting according to level of vulnerability. Using mathematical modelling in Kenya 7, Steen *et al*. found that reaching only high activity FSW with interventions had similar population impact to reaching all FSW.

Another data gap relates to behaviour of men, specifically those who have high numbers of condomless sex partners. We have little knowledge of what types of partnerships these are, what proportion are transactional, including what proportion are with self‐identified FSW.

## 5 Conclusions

Prevention efforts need to be intensified if new HIV infections are to be reduced to 2030 targets. Modelling suggests that “eliminating” HIV transmissions through commercial sex by maximizing engagement with treatment and prevention services would have considerable impact on the number of new infections in Zimbabwe over the next 12 years and beyond. Further work to refine the model and explore impact and cost effectiveness of various targeting approaches is planned. We present evidence that it is possible to attain high uptake and coverage, but that this is dependent on extent and sustainability of resources. Regular, risk‐differentiated, individual peer support through microplanning coupled with community empowerment has the potential to optimize service use to maximize programme impact.

## Competing interests

No competing interests to declare.

## Authors’ contributions

F.C. drafted the paper. S.C. and S.M. undertook analyses of respondent driven sampling surveys and of programme data. E.F. and C.D. provided statistical support. T.M. oversaw programme implementation. L.B.M. undertook modelling. J.B., R.S., O.M. and J.H. provided insights into analyses. L.M.B., V.C. and A.P. devised HIV synthesis model. All authors have been integral to design of studies included here and programme data collection. All authors commented on manuscript draft.
